# Mechanism, frequency, transfusion and outcome of severe trauma in coagulopathic paediatric patients

**DOI:** 10.1007/s00068-020-01398-x

**Published:** 2020-05-24

**Authors:** Arne Driessen, Arasch Wafaisade, Rolf Lefering, Filippo Migliorini, Matthias Fröhlich, Dariusch Arbab, Marc Maegele, Manuel Mutschler

**Affiliations:** 1 Department of Orthopaedic Surgery, University Hospital RWTH Aachen, RWTH Aachen University, Pauwelsstraße 30, 52074 Aachen, Germany; 2 Department of Orthopaedic Surgery, Trauma Surgery and Sports Traumatology, University of Witten/Herdecke, Cologne-Merheim Medical Centre (CMMC), Cologne, Germany; 3 Institute for Research in Operative Medicine (IFOM), University of Witten/Herdecke, Cologne Merheim Medical Centre (CMMC), Cologne, Germany; 4 Department of Orthopaedic Surgery, Klinikum Dortmund, Dortmund, Germany; 5 Department of Foot and Ankle Surgery, Johanniter Waldkrankenhaus Bonn, Bonn, Germany

**Keywords:** Coagulopathy, Haemorrhage, Paediatric trauma, Transfusion, Mortality

## Abstract

**Purpose:**

Acute traumatic coagulopathy can result in uncontrolled haemorrhage responsible for the majority of early deaths after adult trauma. Data on the frequency, transfusion practice and outcome of severe trauma haemorrhage in paediatric patients are inconsistent.

**Methods:**

Datasets from paediatric trauma patients were retrieved from the registry of the German trauma society (TR-DGU^®^) between 2009 and 2016. Coagulopathy was defined by a Quick’s value < 70% (INR (international normalized ratio) > 1.4) and/or thrombocytes ≤ 100 k upon emergency room admission. Children were grouped according to age in 4 different groups (A: 1–5, B: 6–10, C: 11–15 and D: 16–17 years). Prevalence of coagulopathy was assessed. Demographics, injury severity, haemostatic management including transfusions and mortality were described.

**Results:**

5351 primary admitted children ≤ 17 years with an abbreviated injury scale (AIS) ≥ 3 and complete datasets were included. The prevalence of coagulopathy was 13.7% (733/5351). The majority of the children sustained blunt trauma (more than 90% independent of age group) and a combination of traumatic brain injury (TBI) and any other trauma in more than 60% (A, C, D) and in 53.8% in group B. Coagulopathy occurred the most among the youngest (A: 18.2%), followed by all other age groups with approximately 13%. Overall mortality was the highest in the youngest (A: 40.9%) and among the youngest patients with traumatic brain injury (A: 71.4% and B: 47.1%). Transfusion of packed red blood cells (pRBCs) and fresh frozen plasma (FFPs) occurred almost in a 2:1 ratio (or less) across all age subgroups.

**Conclusion:**

Traumatic haemorrhage in association with coagulopathy and severe shock is a major challenge in paediatric trauma across all age groups.

**Electronic supplementary material:**

The online version of this article (10.1007/s00068-020-01398-x) contains supplementary material, which is available to authorized users.

## Background

Trauma is the leading cause of death in persons aged 1–44 years [1] and accounts for approximately 10% of all deaths in this group [2]. Despite substantial improvements in acute trauma care over the past decades, uncontrolled haemorrhage is still responsible for approximately 50% of all trauma-related deaths within the first 48 h after hospital admission [3, 4]. Meanwhile, a new appreciation of coagulopathy in acute trauma care has evolved and the acute traumatic coagulopathy (ATC) is now recognized as an own entity [5–7]. To date, six key initiators of ATC have been ascribed: tissue trauma, shock, haemodilution, hypothermia, acidemia, and inflammation [8]. Brohi et al. [9] emphasized the role of hypoperfusion and shock for the initiation of ATC. In adult trauma patients, frequencies for ATC upon emergency room (ER) admission have been reported to range between 20 and 60% according to definition [5, 10, 11]. However, data on the incidence for coagulopathy and shock in the paediatric population with predominantly blunt injury are rare. One large report including both shock and coagulopathy measures has been published for children with mostly penetrating injury [12]. Hendrickson and colleagues have evaluated coagulopathy in a single centre cohort of 102 children with > 80% blunt injuries on ER presentation and quantified the relationship with mortality [13]. We report on the frequency of coagulopathy upon ER admission in children (≤ 17 years) with predominantly blunt injury, subsequent on transfusion practice in cases of severe trauma haemorrhage and on outcome based upon data derived from the trauma registry of the Deutsche Gesellschaft für Unfallchirurgie (German Trauma Society/TR-DGU^®^).

## Methods

**Fig. 1:**
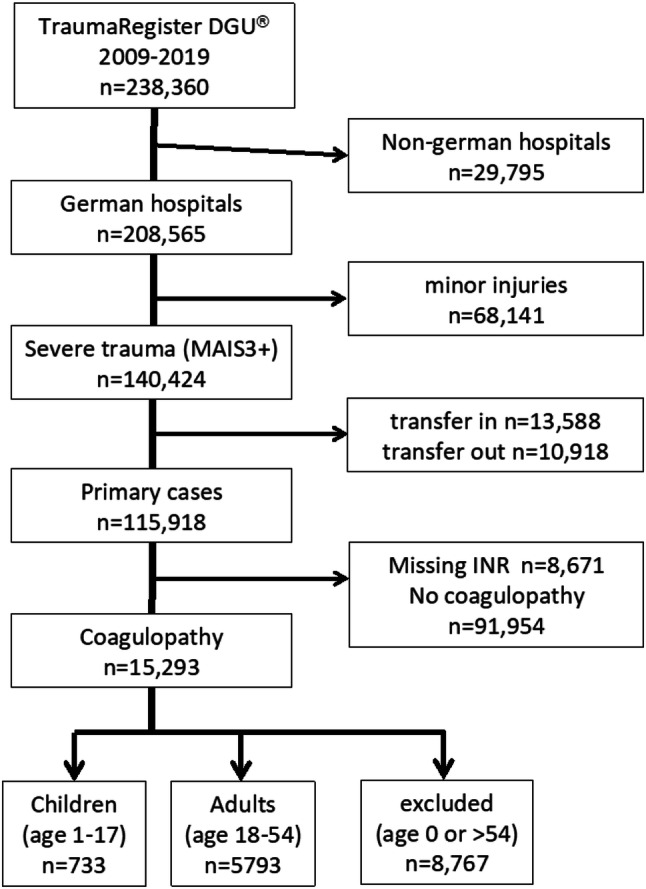
Flowchart of patient selection according to the eligibility criteria

Over 230,000 trauma patients that have been entered into the TR-DGU^®^ database between 2009 and 2016 were reviewed. 733 datasets from children aged 1–17 years were identified for further analysis (Fig. 1). All children had sustained significant trauma (Abbreviated injury Scale—AIS ≥ 3), had been primarily admitted and had a complete dataset for Quick’s value [prothrombin time ratio (PTr)], PTT (partial thromboplastin time) and number of thrombocytes upon ER admission. For further analysis, this population was divided into four subgroups according to age: group A: age 1–5, group B: age 6–10, and group C: age 11–15 and group D: age 16–17 years. Subgroups were compared to control group E age 18–54 (*n* = 5793).

### The TR-DGU^®^ (Trauma Register-Deutsche Gesellschaft für Unfallchirurgie)

The TR-DGU^®^ was founded in 1993 by the German Trauma Society [14]. It is a prospective, multicentre, standardized and pseudonymous documentation of multiple injured trauma patients at four consecutive post-trauma phases from initial injury to hospital discharge: (1) the pre-hospital phase; (2) emergency room and initial surgery; (3) intensive care unit (ICU) and (4) outcome status at discharge and description of injuries and procedures. Detailed information on demographics, injury pattern, co-morbidities, pre-hospital and early in-hospital management, time course, relevant laboratory findings, and outcome of each individual are captured. Between 2009 and 2016, over 50,000 patients from more than 400 hospitals had been entered into the database. All injuries are coded using the Abbreviated Injury Scale (AIS) [15]. Scientific data analysis is approved according to a peer-review procedure laid down in the publication guideline of TR-DGU^®^.

The present study is in line with the publication guidelines of the TR-DGU^®^ and registered as TR-DGU project ID 2018-005.

All methods were carried out in accordance with relevant guidelines and regulations. More information can be found online under https://www.traumaregister-dgu.de.

### Definition of coagulopathy and magnitude of trauma haemorrhage

Coagulopathy was defined by the presence of a Quick’s value (prothrombin time ratio/PTr) < 70% upon ER admission [16]. The prothrombin time test was first introduced by A. J. Quick in 1935 and is either expressed in Quick-% (70–130% = normal) or as a prothrombin time ratio (PTr) [16]. Although the international normalized ratio (INR) was introduced as a coagulation test, most German physicians and medical institutions prefer the prothrombin time in Quick (%). Thus, the TR-DGU^®^ documents prothrombin time in Quick-%. A Quick’s value of < 70% is equivalent to an INR (international normalized ratio) of approximately 1.4. Coagulopathy was distinguished via laboratory findings: Quick’s value < 70% (INR > 1.4) or PTT ≥ 40 or thrombocyte count of ≤ 100,000. Demographics, injury severity, haemostatic management including volume administration and transfusions and outcome were assessed in all groups.

### Statistical analysis

All data were analysed by SPSS statistical software (version 24, IBM Inc., Armonk, NY). Clinical data were compared using the Chi^2^-test for categorical variables and the Mann–Whitney *U* test for continuous variables. A *P* value < 0.05 was considered significant.

## Results

A total of 238,360 patient recordings entered into the TR-DGU^®^ between 2009 and 2016 were identified for further analysis. Coagulopathy upon Emergency Department (ED) admission was present in 733 out of 5351 (13.7%) children matching the criteria (Fig. 1).

The vast majority of injured children were male with increasing percentage in the higher age groups (A: 57.3%; B: 61.5%; C: 71.1%; D: 74.7%). Furthermore, in all age groups well above 90% of children suffered injuries caused by blunt trauma. The majority of elderly children in group D suffered traffic related injuries in motorcycle accidents (37.0%) and car accidents (25.7%). Likewise, the majority of children in other age groups suffered injuries caused by car accidents (A: 23.8%; B: 27.8%; C: 22.3%) followed by bicycle injuries (especially group C: 20.8% and B: 12.2%). More than 21% of the youngest children (group A) were injured by high falls (> 3 m) followed by 17.8% (group C), 11.3% (group D) and 9.6% (group B). Demographics and trauma mechanism according to the age groups of children in coagulopathy are depicted in Table 1.

**Table 1:** Demographics and injury pattern of children admitted to the emergency department (ED) in the state of coagulopathy according to different age groups

Age group	A (1–5 years)	B (6–10 years)	C (11–15 years)	D (16–17 years)	E control
Children with coagulopathy (ChiCo)	*n* = 110	*n* = 117	*n* = 205	*n* = 301	*n* = 5793
	733/5351 (13.7%)				
Male sex	57.3% (63)	61.5% (72)	71.1% (145)	74.7% (224)	75.9% (4369)
Mechanism of trauma					
Blunt trauma	97.0% (98)	94.7% (107)	93.5% (187)	92.3% (262)	92.2% (5094)
Cause of injury					
Car accident	23.8% (25)	27.8% (32)	22.3% (45)	25.7% (75)	32.8% (1864)
Motorbike	0% (0)	0.9% (1)	5.9% (12)	37% (108)	18.9% (1076)
Bicycle	1.9% (2)	12.2% (14)	20.8% (42)	4.5% (23)	4.2% (283)
Pedestrian	29.5% (31)	31.3% (36)	20.3% (41)	8.2% (24)	18.5% (132)
High fall (> 3 m)	21% (22)	9.6% (11)	17.8% (36)	11.3% (33)	18.4% (1044)
Low fall (< 3 m)	7.6% (8)	4.3% (5)	1.0% (2)	0.3% (1)	5.5% (310)
Others	16.2% (17)	13.9% (16)	11.9% (24)	13% (38)	13.4% (762)

### Injury severity and injury pattern

When compared to all trauma patients (E: 51.8%) recorded in the TR-DGU^®^, the AIS head in all age groups is the highest in the youngest (A: 80%) decreasing in other age groups to the oldest in group D (55.8%) (Table 2). In contrast, the percentage of patients with thoracic injuries (A: 38.3% to D: 63.1%) and extremity injuries (A: 20.9% to D: 53.8%) is increasing steadily with growing age reaching almost the average percentage of the control group. More than 50% of the patients aged between 1 and 10 years suffered a monotrauma but only 19.2% of group A and 14.5% of group B suffered an isolated brain injury. Over 60% of the patients in age group A, C and D and over 50% in group B suffered a combined trauma (head plus other body region).

**Table 2:** Injury severity, injury pattern, hypotension and transfusion rate according to age groups

Age group	A (1–5 years)	B (6–10 years)	C (11–15 years)	D (16–17 years)	E control
Children with coagulopathy (ChiCo)	*n* = 110	*n* = 117	*n* = 205	*n* = 301	*n* = 5793
	733/5351 (13.7%)				
Injury severity					
GCS (mean)	6.7 (100)	8.5 (111)	7.9 (191)	8.2 (291)	8.97 (5506)
ISS	30.2 (110)	27.3 (117)	32.6 (205)	35.5 (301)	33.3 (5793)
Relevant injuries (Abbreviated Injury Scale (AIS) > 3) (percent of total {n})					
AIS head	80.0% (88)	65.8% (77)	68.8% (141)	55.8% (168)	51.8% (3001)
AIS thorax	38.3% (42)	39.3% (46)	58.5% (120)	63.1% (190)	64.8% (83,754)
AIS abdomen	14.5% (110)	28.2% (33)	24.9% (51)	34.9% (105)	30.0% (1736)
AIS extremities	20.9% (23)	34.2% (40)	37.6% (77)	53.8% (162)	50.6% (2930)
Injury pattern					
No head injury	54.5% (60)	51.3% (60)	36.1% (74)	31.6% (95)	33.6% (1946)
Isolated head injury	19.2% (21)	14.5% (17)	7.3% (15)	2.7% (8)	6.3% (365)
Combined trauma	63.6% (70)	53.8% (63)	64.4% (132)	60.5% (182)	54.9% (3183)
Transfusion rate					
pRBC	35.8% (39)	30.8% (36)	37.1 (75)	42.2% (125)	44.8% (2574)
FFP	18.3% (20)	18.8% (22)	24.3% (49)	29.4% (87)	31.% (1805)
Transfusion rate pRBCs without TBI (traumatic brain injury)					
pRBCs without TBI (% of total in age group) (*n*)	37.1% (33/89)	36% (36/100)	29% (73/187)	43.1 (124/288)	46.5% (2501/5382)

*GCS* Glasgow Coma Scale, *ISS* Injury Severity Score, *pRBCs* packed red blood cells, *FFP* fresh frozen plasma, *TBI* traumatic brain injury)

### Coagulopathy

Within the age groups, coagulopathy occurred the most among the youngest (A: 18.2%), followed by all other age groups with approximately 13%. The mean INR for children in group A was 1.92 (± 1.25), group B 1.82 (± 1.24), group C 1.79 (± 0.87), group D 2.07 (± 1.66) and control group 1.92 (± 1.35). A total of 733 children presented with a Quick’s value < 70% (INR > 1.4) or PTT ≥ 40 or thrombocyte count of ≤ 100,000 (Table 3). The majority of these children can be found among the youngest (A: 57.3%, *n* = 63). Groups B–D are depicted in Table 3.

**Table 3:** Outcome and mortality depending on injury pattern of children admitted to the ED (emergency department)

Age group	A (1–5 years)	B (6–10 years)	C (11–15 years)	D (16–17 years)	E control
	733/5351 (13.7%)				
Children with coagulopathy (% per group)	18.2% (*n* = 110)	13.4% (*n* = 117)	12.7% (*n* = 205)	13.3% (*n* = 301)	10.9% (*n* = 5793)
Hospital stay					
Length of hospital stay (days)	14.1 (SD 14.7)	14.8 (SD15.0)	17.2 (SD 20.9)	18.9 (SD 18.6)	22.6 (SD 25.1)
ICU stay (days)	8.0 (SD 11.2)	8.0 (SD 13.1)	8.7 (SD 10.6)	10.7 (SD 13.6)	11.4 (SD 14.7)
Ventilator time (days)	4.5 (SD 2.0)	4.42 (SD 8.1)	4.6 (SD 6.7)	6.3 (SD 10.5)	6.8 (SD 11.2)
Overall outcome (percent of total {*n*})					
Overall mortality	40.9% (45)	29.1% (34)	33.2% (68)	31.9% (96)	32.2% (1867)
Mortality with TBI	48.9% (43/88)	41.6% (32/77)	43.3% (61/141)	46.4% (78/168)	47.4% (1421/3001)
Mortality without TBI	9.1% (2/22)	5% (2/40)	10.9% (7/64)	13.5% (18/133)	16% (446/2792)

Children with coagulopathy (ChiCo) (INR ≥ 1.4; PTT ≥ 40; thrombocytes ≤ 100 k) according to age groups, hospital stay and mortality

*ICU* intensive care unit, *TBI* traumatic brain injury

### Management

**Fig. 2:**
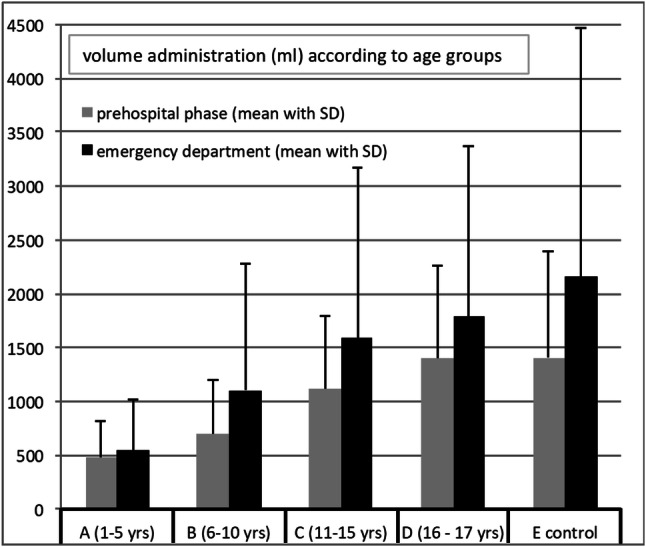
Volume administration during prehospital resuscitation in ED (emergency department) depending on age groups

**Fig. 3:**
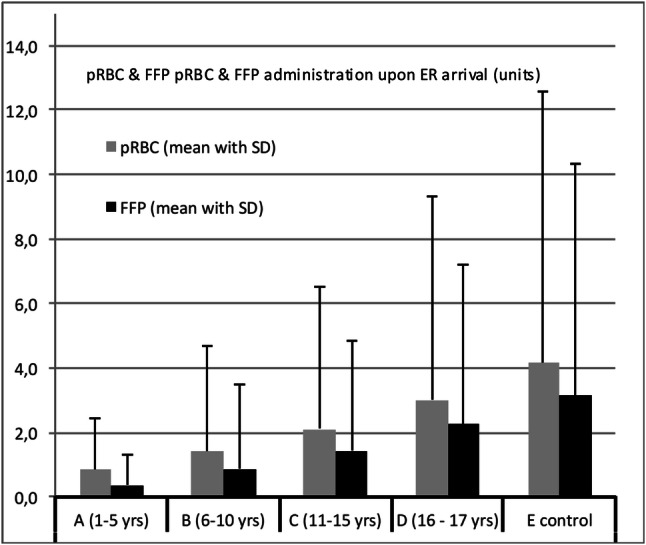
Transfusion requirements for pRBCs (packed red blood cells) and FFP (fresh frozen plasma) until ICU (intensive care unit) admission during the prehospital resuscitation and in the ED (emergency department) according to different age groups

The length of hospital stay (days) is increasing with age (A: 14.1, B: 14.8, C: 17.2, D: 18.9) as well as the time on ICU (A: 8.0, B: 8.0, C: 8.7, D: 10.7) and quantity of days patients depend on mechanical ventilation (A: 4.5, B: 4.4, C: 4.6, D: 6.3). Transfusion requirements until ICU admission and volume administration during the prehospital resuscitation and in the ED are depicted in Figs. 2 and 3. The total amount was the smallest in the youngest and decreases with age reflecting an age (and body weight)-based volume substitution.

On average and including those without transfusion, children in group A received less than one pRBC (0.86 ± 1.55) and FFP (0.37 ± 0.911), children in group B received 1.4 ± 3.26 pRBCs and 0.89 ± 2.59 FFP rather reflecting a 2:1 transfusion rate. This ratio of pRBCs to FFP is converging to a more balanced administration in group C (2.12 ± 4.39–1.45 ± 3.38) and D (3.01 ± 6.34–2.27 ± 4.94). The overall transfusion rate for pRBCs and FFPs is the highest in group D (42.2%, 29.4%) besides more than 30% for pRBCs and approximately 20% for FFPs in all other age groups (Table 2). As expected, transfusion rates of pRBCs were lower and independent of age in the groups having sustained only TBI than in those having sustained any other injury but TBI.

## Discussion

The aim of this study was threefold: (1) to determine the mechanism of injury upon ER admission in traumatically injured children of different age groups, (2) to analyse frequency of coagulopathy and transfusion practice in those children when stratified according coagulopathy and (3) to analyse outcomes of these paediatric trauma patients.

Regarding the demographical results, the majority of the age groups of 16 and 17 years suffered traffic-related injuries during car or motorbike accidents which can be understood as a higher risk being involved in accidents shortly after the driving license was issued. With growing age, the percentage of male children suffering from coagulopathy is increasing indicating a higher affinity of boys to risky recreational activities.

The data allow a distinction between car accidents as passenger of the vehicle and pedestrians. About one-third of children in groups A and B were injured as pedestrian. Remarkably high falls range from 9.6% (B, *n* = 11) to 21% (A, *n* = 22) and, therefore, are quite common in all age groups but with more than 1/5 fatally among the youngest. Assuming these numbers are referring back to defenestration and falls from balcony, these events could easily be prevented by focused precautions.

As expected, relevant head injuries are decreasing with growing age; thoracic injuries and injuries of the extremities are increasing with growing age reflecting the importance of weight and surface of the head depending on age and upgrowth of children. Nevertheless, isolated brain trauma is more often in the youngest but still the commonest in combination with other injuries possibly explaining children’s coagulopathy. In addition, lacerations at the head can cause relevant blood loss resulting in coagulopathy especially in the youngest.

Thus, irrespective of age, the majority of children in coagulopathy sustained a combination of brain trauma plus any other injury.

In this paediatric trauma population, which comprised 5351 injured children entered into the TR-DGU^®^ in the period from 2009 to 2016, coagulopathy was present in 13.7% (*n* = 733). Hendrickson and colleagues have evaluated coagulopathy also in a cohort of civilian paediatric trauma patients with > 80% blunt trauma [13]. In their single-centre cohort of 102 children (mean age 6 years; mean ISS 22 points), an abnormal prothrombin time was found in 72% while the PTT was abnormal in 38%.

In a previous study, a frequency of 27% was reported from a cohort of children admitted to U.S. combat hospitals in Iraq and Afghanistan [17]. The majority of these children had sustained a penetrating trauma due to explosive devices (43%) and gunshots (26%), while children in the present study had mainly sustained a blunt trauma mechanism (> 90%) in all age groups. Taken together, both studies indicate that the frequency of trauma-associated coagulopathy and shock in children appears to be slightly lower compared to the adult trauma population where in both blunt and penetrating trauma patients coagulopathy is present in approximately one out of three trauma patients [11, 18]. Further studies in adult trauma cohorts have reported even higher frequencies, but depending on definition and study design [8, 10, 19].

The definition of shock used for adults within the TR-DGU RRsyst < 90 mmHg is not applicable in young children which is why shock as an outcome parameter could not be analysed in the present study. But literature, e.g. Patregnani et al. [17] reported 46% to be in severe shock and 38% being coagulopathic upon ER admission in their paediatric trauma cohort with an ISS ≥ 15. In children with an ISS < 15, coagulopathy and shock were only present in 20% and 33%, respectively. The mean ISS in the study by Hendrickson et al. [20] was along with a higher frequency of abnormal standard coagulation parameters (PT, PTT, fibrinogen, haemoglobin, and platelet count) upon ER admission. The association between injury severity and coagulopathy is also well known in adult trauma population and trauma load has been identified as an important risk factor for coagulopathy and shock [5, 11, 18, 19, 21].

As previously shown, the presence of coagulopathy and severe shock was also associated with increased overall mortality [5, 11, 17, 18]. In the present study, one out of three children with coagulopathy died after admission. Several authors have previously identified coagulation abnormalities upon ER admission as an independent predictor of prognosis and mortality [22, 23]. Hendrickson et al. [13] reported abnormal prothrombin, partial thromboplastin times and platelet counts to be strongly associated with mortality in their cohort of severely blunt injured paediatric patients (*p* = 0.005; *p* = 0.001, and *p* < 0.001) remaining significant even after multivariate analysis and adjusting for injury severity (ISS). Furthermore, uncontrolled haemorrhage is considered to be responsible for approximately 50% of all trauma-related in-hospital deaths within the first 48 h of admission [3, 4], but heavily shifted towards the first 2 h [24, 25] emphasizing the importance of early recognition of coagulopathy and thus prompting early goal-directed therapy.

In paediatric trauma patients, special considerations have to be made regarding volume substitution. All estimates of blood volume, volume loss and replacement are based on weight with children over 3 months having an estimated blood volume of 70 ml/kg body weight and younger infants having an estimated 90 ml/kg body weight [26–28].

During pre- and early in-hospital care, patients in the state of severe shock and coagulopathy received increasing amounts of intravenous fluids. Iatrogenic dilution is often caused by unguided and over-administration of fluids in the acute phase of trauma care, and has been shown to interfere with coagulation and to diminish haemostasis [7, 8, 19, 29]. Furthermore, increasing frequency of coagulopathy and increasing levels of shock have been described in both paediatric and adult trauma populations [8, 11, 17–19, 30, 31]. The presence of shock and the resulting hypoperfusion lead to acidemia which itself is another mechanism of inducing coagulopathy by interference with coagulation enzyme activity [8, 32–34]. Similar effects have been described in the presence of hypothermia [8, 19, 33].

Besides the early recognition of coagulopathy and severe shock, early initiated goal-directed transfusion management is crucial [35, 36]. In the present study, 30–40% coagulopathic children received blood products independent of age. In general, pRBCs and FFPs were transfused at a 2 (or less):1 ratio across all age subgroups. In the adult trauma population, high plasma to pRBC ratios have been shown to be associated with improved survival, primarily by decreasing death from haemorrhage both in military and civilian settings [37, 38].

Interestingly, a retrospective study of patients receiving FFP alone compared to patients receiving coagulation factor concentrates without FFP showed the patients receiving FFP alone had an increased frequency of multi-organ failure [39].

However, the implementation of a massive transfusion protocol with a fixed 1:1 ratio did not improve overall mortality in a small under-powered single-centre cohort of paediatric patients [20]. However, MT occurs less common in children due to their greater physiological reserve and tolerance to blood loss. Due to varying body volumes by age, gender and weight, defining clear criteria for MT in children is difficult. Chidester and colleagues observed in their single-centre study that mortality was not significantly different between children who received massive transfusion protocol and children who were given blood at the physician’s discretion [40].

Larger studies are essential to determine the potential benefit of this approach, which has been observed frequently in adult trauma patients. Moreover, the implementation of management and transfusion guidelines comparable to current guidelines for adult trauma patients is also needed for the paediatric population [41].

Certain limitations of this investigation have to be acknowledged. This is a retrospective study with all the associated shortcomings, for example, the introduction of a selection bias. Furthermore, a significant number of children had to be excluded from the present analysis, either due to missing INR and Quick’s value or secondary admission. Furthermore, hypothermia has been shown to be an important trigger of coagulopathy [8, 19, 25]. Unfortunately, body temperature is only poorly documented in the TR-DGU^®^ and, therefore, the role of hypothermia could not be substantiated in the present analysis. Additionally, the vast majority of our patients suffered from blunt trauma, as penetrating trauma is rare (less than 10%) in German trauma populations. Further prospective trials are needed to gain knowledge on the physiology and pathophysiology of severe shock and coagulopathy in paediatric trauma patients. Additional studies are required to determine if damage control resuscitation strategies improve survival in paediatric populations.

## Conclusion

The frequency of coagulopathy upon ER admission in the paediatric trauma population was 13.7%. Blood products (pRBC and FFP) were transfused in a close to 2:1 ratio across all age subgroups. One-third or more (group A) of coagulopathic children died after hospital admission across all age subgroups. Depending on the injury pattern, mortality with TBI only was the highest. Prospective studies are warranted to determine if early and more aggressive coagulation management including factor replacement and the implementation of (massive) transfusion protocols (MTPs) may improve outcomes in this population.

## Electronic supplementary material

Below is the link to the electronic supplementary material.

Supplementary file1 (PDF 931 kb)

## Data Availability

All data analysed for this study are managed by the TraumaRegister-DGU^®^. The scientific steering group is the Committee on Emergency Medicine, Intensive Care and Trauma Management of the German Trauma Society (Sektion NIS). Data analysis is approved according to a peer-review procedure established by Sektion NIS. Please find more information https://www.traumaregister-dgu.de. TraumaRegister-DGU^®^ is setting global standards for the quality management of severely injured. Almost 700 hospitals from 9 different countries are participating. Mostly the participating hospitals are located in Germany, but other countries such as Belgium, Finland, Luxembourg, the Netherlands, Austria, Switzerland, Slovenia and United Arab Emirates are contributing increasingly. Since its founding in 1993, data from more than 270,000 cases have been documented. In 2017 alone, about 35,000 cases have been entered in TraumaRegister-DGU^®^. For the hospitals, TraumaRegister-DGU^®^ is not only an instrument for external quality assurance, but has also provided a basis for clinical and healthcare research for years.

## Abbreviations

AIS: Abbreviated Injury Scale
ATC: Acute traumatic coagulopathy
BE: Base excess
BP: Blood pressure
ChiCo: Children in coagulopathy
DGU: Deutsche Gesellschaft für Unfallchirurgie (German Trauma Society)
ED: Emergency department
FFP: Fresh frozen plasma
GCS: Glasgow Coma Scale
ICU: Intensive care unit
INR: International normalized ratio
ISS: Injury Severity Score
MOF: Multiple organ failure
MTP: Massive transfusion protocol
NISS: New Injury Severity Score
PH: Prehospital
pRBC: Packed red blood cells
PTr: Prothrombin time ratio
PTT: Partial thromboplastin time
RISC: Revised Injury Severity Classification
SOFA: Sequential organ failure assessment
TASH: Trauma-Associated Severe Haemorrhage Score
